# Single Locus Controls Majority of Armor Evolution in Two Populations of Sticklebacks

**DOI:** 10.1371/journal.pbio.0020143

**Published:** 2004-05-11

The astounding diversity of life—different body shapes and sizes, physiologies, and behaviors—stems from the accumulation of genetic changes through the process we call evolution. But catching a glimpse into the process of evolution at the gene level is difficult, mostly because significant changes to the plant and animal species of today happened a long time ago. Nevertheless, biologists are keen to understand exactly how evolution progresses. For example, how many genes must be altered before noticeable shifts in appearance can be seen? Is evolution the result of changes in many genes with small additive effects, or of just a few mutations that exert a strong influence?[Fig pbio-0020143-g001]

**Figure pbio-0020143-g001:**
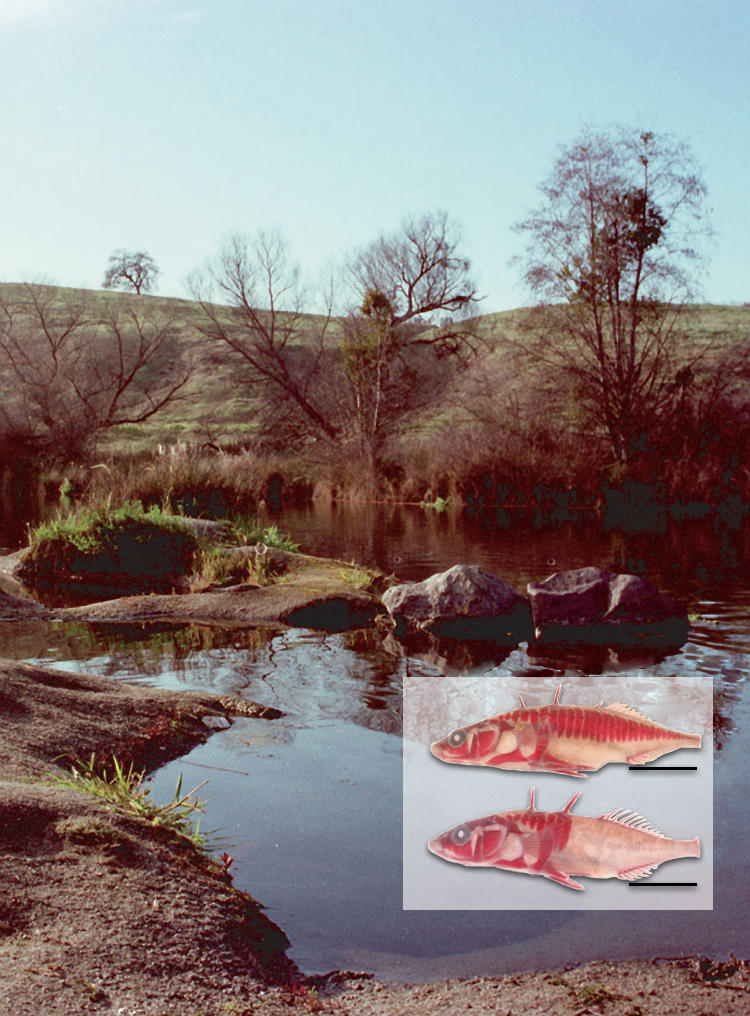
Complete and low armor stickleback morphs, Friant, California

To tackle these questions, Pamela Colosimo and colleagues turned to threespine stickleback fish, a longtime favorite model system of evolutionary biologists because of its relatively youthful evolutionary history. At the end of the last ice age 10,000 years ago, when glaciers all over the Northern Hemisphere began to melt, small populations of these originally marine-dwelling fish became trapped in newly formed lakes. There, isolated stickleback colonies adapted to new ecological conditions—different predators, food availability, water chemistry, and temperature—and now look distinctly different from their marine ancestors. One of the most obvious changes in appearance is in their body armor—they come in three distinct types, or “morphs.” Marine sticklebacks are covered from head to tail with rows of tightly packed boney plates (a complete morph), while those found in freshwater lakes have fewer body plates (a partial morph) or almost none at all (a low morph). Colosimo and colleagues found that a single region of the genome is largely responsible for the dramatic changes in plate morph, and that this is true for two widely separated populations of independently evolving freshwater sticklebacks.

To uncover the genomic regions that affect armor, Colosimo's team crossed fully armored marine sticklebacks from Japan with deep-water, or benthic, low morph fish from Paxton Lake in British Columbia, Canada. They then “mapped” the full genome of second generation offspring using 160 known genetic markers, or loci, as guideposts for distinct regions of the genome—loci that are inherited along with differences in the overall type of plating, and individual plate number and size.

The team found that one such locus explained 75% of the variation in plate morphs. Offspring that carried two alleles—versions of the gene—from their marine grandparents, genotype *AA,* were almost always fully plated. Those that inherited two copies of the allele from their benthic progenitors, *aa,* were mostly low morphs with very little plating. And *Aa* heterozygous fish (with one allele from each population) had mostly full or partial plates. Colosimo and colleagues also found three other regions in the genome that significantly affected the number and size of plates. These modifiers had an additive effect—the more benthic alleles inherited, the fewer and smaller the plates; more marine alleles caused a trend toward greater armor.

But is this genetic architecture the same for every independently evolving population of lake-bound sticklebacks in North America? Or did the geographically isolated freshwater groups loose their plates through mutations in different genes? Colosimo and colleagues mapped the genome of a population of sticklebacks from Friant, California, which is 800 miles away from Paxton Lake, and found that the same major locus seemed to be controlling plate morph there as well. Crossing a low morph from Friant with a low morph from Paxton yielded only offspring with very little armor. Further, some of the modifiers uncovered in the Paxton fish were also acting on the Friant sticklebacks. So, though these two populations of fish have been separated for 10,000 years, loss of armor in both groups probably stemmed from changes in the same genetic pathway.

Without knowing the precise sequence of these genes, it is impossible to tell exactly how and when the alleles that reduce armor arose. Small numbers of individuals with genes causing less plating could have been present in ancestral populations of marine sticklebacks when they were originally locked in newly formed lakes. Alternatively, reduced armor could have arisen independently in different lakes following isolation if, for example, some genes that control armor are predisposed to mutation, or certain armor-related mutations are more advantageous than others. But however it happened, this study clearly shows that dramatic morphological evolution can result from a small number of genetic changes. Further study of this classic system should provide a detailed picture of the genes involved, and of the molecular events that underlie morphological changes in natural populations evolving in new environments.

